# Genetics of vascular dementia – review from the ICVD working group

**DOI:** 10.1186/s12916-017-0813-9

**Published:** 2017-03-06

**Authors:** M. Arfan Ikram, Anna Bersano, Raquel Manso-Calderón, Jian-Ping Jia, Helena Schmidt, Lefkos Middleton, Benedetta Nacmias, Saima Siddiqi, Hieab H.H. Adams

**Affiliations:** 1 Department of Epidemiology, Erasmus MC, Rotterdam, The Netherlands; 2 Department of Radiology and Nuclear Medicine, Erasmus MC, Rotterdam, The Netherlands; 3 Department of Neurology, Erasmus MC, Rotterdam, The Netherlands; 4 Cerebrovascular Unit IRCCS Foundation Neurological Institute C. Besta, Milan, Italy; 5 Department of Neurology, University Hospital of Salamanca, Salamanca, Spain; 6 Institute of Biomedical Research of Salamanca (IBSAL), University of Salamanca-CSIC-SACYL, Salamanca, Spain; 7 Department of Neurology, Xuan Wu Hospital, Capital Medical University, Beijing, China; 8 Department of Neurology, Medical University of Graz, Graz, Austria; 9 Neuroepidemiology and Ageing Research Unit, School of Public Health, Imperial College London, London, UK; 10 Department of Neuroscience, Psychology, Drug Research and Child Health, University of Florence, Florence, Italy; 11 IBGE, Islamabad, Pakistan; 12 Department of Epidemiology, Erasmus MC University Medical Center, Wytemaweg 80, 3015 CN Rotterdam, The Netherlands

**Keywords:** Vascular dementia, Cerebral small vessel disease, Genetics, Magnetic resonance imaging

## Abstract

**Background:**

Vascular dementia is a common disorder resulting in considerable morbidity and mortality. Determining the extent to which genes play a role in disease susceptibility and their pathophysiological mechanisms could improve our understanding of vascular dementia, leading to a potential translation of this knowledge to clinical practice.

**Discussion:**

In this review, we discuss what is currently known about the genetics of vascular dementia. The identification of causal genes remains limited to monogenic forms of the disease, with findings for sporadic vascular dementia being less robust. However, progress in genetic research on associated phenotypes, such as cerebral small vessel disease, Alzheimer’s disease, and stroke, have the potential to inform on the genetics of vascular dementia. We conclude by providing an overview of future developments in the field and how such work could impact patients and clinicians.

**Conclusion:**

The genetic background of vascular dementia is well established for monogenic disorders, but remains relatively obscure for the sporadic form. More work is needed for providing robust findings that might eventually lead to clinical translation.

## Background

While vascular dementia (VaD) is the second most common form of dementia [1], studies on its genetic basis are scarce. It is well established that the risk of VaD can be modified by various lifestyle factors, physiological risk factors, and comorbidities [1]. However, its genetic component remains poorly understood, especially when compared to other causes of dementia such as Alzheimer’s disease (AD), Parkinson’s disease, and frontotemporal lobar degeneration. Only a single study in 24 twins has attempted to determine the heritability of VaD, but was unable to identify a significant genetic component [2]. While this could suggest that the environment plays a larger role, a careful interpretation is warranted given the limited sample size and the heterogeneity in VaD definitions. Furthermore, several lines of evidence actually indicate that VaD might have a substantial genetic component.

In this review, we summarize the current knowledge of the genetics of VaD and provide an overview of future developments in genetic research on VaD. Besides the diagnosis of VaD, we extend our report to include several associated phenotypes, including AD and stroke, as well as cerebral small vessel disease (CSVD), which can be considered as a precursor or a distinct form of VaD (see dedicated consensus report in this volume [3]). These have all been shown to be significantly heritable and to potentially share predisposing genetic factors.

This review builds on the 9th International Congress on Vascular Dementia (ICVD), which took place in Ljubljana, Slovenia, on 16–18 October 2015. The ICVD meeting was structured in several Working Groups that focused on specific topics within the field of VaD. The current report is based on the work of the ‘Genetics of Vascular Dementia’ Working Group.

Here, we provide a narrative review on the genetic aspect of VaD. First, we provide an overview of monogenic disorders that results in VaD. Second, we review our current knowledge of the genetic background of sporadic VaD, which remains obscure. Third, we point to future developments in the field that are likely to shed light on the role of genetics. Finally, we discuss how a proper understanding of genetics can have clinical implications for VaD.

## Review

### Monogenic disorders

The most apparent evidence for a genetic basis of VaD is the fact that dysfunction of single genes can lead to the development of VaD. Several of these monogenic disorders have been described and are summarized in Table 1, along with their clinical and neuroimaging features relevant to VaD. Below, we highlight the most important monogenic disorders.

**Table 1:** Monogenic disorders associated with cerebral small vessel disease (modified from Bersano et al. [7])

Disease	CADASIL	Fabry disease	RVCL	COL4A1	CARASIL
OMIM	#125310	#301500	#192315	#120130	#60142
Pattern of inheritance	Autosomal dominant	X-linked recessive	Autosomal dominant	Autosomal dominant	Autosomal recessive
Gene	*NOTCH3*	α-GAL A gene (GLA)	TREX1	COL4A1	HTRA1
Locus	19p13	Xq22	3p21.3-p21.2	13q34	10q25
Gene product	Notch 3 receptor	Alpha galactosidase A enzyme	DNA specific 3’-5’ exonuclease DNase III	Type IV collagen α1	HTRA1 serine peptidase/protease 1
Clinical manifestations					
Stroke					
Age at onset, years	20–70	33–46 (M)/40–52 (F)	40–50	14–49	20–40
Stroke subtype					
Small vessel disease	+	+	+	+	+
Large vessel disease	–	+	–	–	–
Cardioembolic	–	+	–	–	–
Hemorrhagic	Rare	Rare	–	+	–
Other neurological manifestations					
Psychiatric disturbance	+	+	+	+	+
Migraine with/without aura	+	–	+	+	–
Seizures	+		+	+	
Cognitive impairment	+	±	+	+	+
Extra-neurological manifestations					
Neuropathy	–	+ (80%)	±	+	–
Myopathy	–	–	+	+	+
Renal disease	–	+	+	+	–
Skin involvement	–	+	–	–	–
Ocular involvement	± Retinal arteriolar narrowing	+ Cornea verticillata	+ Retinopathy	+ Cataracts, retinopathy	+ Retinopathy
Gastrointestinal involvement	–	+	±	+	–
Cardiac involvement	–	+	–	+	–
Others		Acroparhestesia, hypoacusia	Raynaud phenomena (80%), hepatopathy	Poroencephaly, prenatal bleeding, infantile hemiparesis	Alopecia, spondylosis deformans, acute lumbago
Radiological findings					
White matter lesions	+	+	+	+	+
Lacunar lesions	+	+	+	+	+
Cortical-subcortical lesions	–	+	±	–	–
Intracerebral hemorrhage (ICH)	+	+	–	+	+
Aneurysms	–	+	–	+	–
Peculiar findings	Temporal lobe hyperintensities, external capsule involvement	Pulvinar hyperintensities on T1-wheighted images	Subcortical contrast-enhancing lesions with surrounding edema	Porencephaly, ICH	–
Pathological findings	Granular osmiophilic material surrounding vascular smooth muscle cells	Cytoplasmatic Gb3 inclusions in vascular endothelial cells and smooth muscle cells	Multilaminated vascular basement membranes	Interruption and thickening of basement membrane	Degeneration in vascular smooth muscle cells

*CADASIL* cerebral autosomal dominant arteriopathy with subcortical infarcts and leukoencephalopathy, *CARASIL* cerebral autosomal recessive arteriopathy with subcortical infarcts and leukoencephalopathy, *RVCL* retinal vasculopathy with cerebral leukodystrophy

A well-known monogenic disorder is cerebral autosomal dominant arteriopathy with subcortical infarcts and leukoencephalopathy (CADASIL), which is the most frequent heritable cause of VaD. It results from mutations of the *NOTCH3* gene, mainly involving the cysteine residue, on chromosome 19q12 that cluster in exons 3 and 4. Common manifestations are migraine headaches, recurrent subcortical ischemic events, cognitive impairment, mood disorders, and seizures [4]. This disorder has been the subject of multiple reviews, and we refer to such an article by Chabriat *et al.* for more information on CADASIL [4].

Another monogenic form, first described in 1989, is Fabry disease (FD) an X-linked lysosomal disease due to a mutation of the *GLA* gene (Xq22), resulting in an absent or reduced a-galactosidase activity that finally leads to accumulation of glycosphingolipid (globotriaosilceramide) in different organs. Stroke or transient ischemic attack, renal disease, and cardiomyopathy have been observed in FD patients [5–7]. FD has been described as a potential cause of young stroke (mostly lacunar but also large vessel and cardioembolic) in 0.4–11% of patients [8, 9].

Other more rarely described single-gene disease entities are the *COL4A1-A2* gene-related arteriopathy, which is a possible cause of small vessel arteriopathy and intracranial hemorrhages [10], and retinal vasculopathy with cerebral leukodystrophy, which now subsumes three previously considered separate entities (cerebroretinal vasculopathy, hereditary endotheliopathy, retinopathy, nephropathy and stroke, and hereditary vascular retinopathy). The typical features of retinal vasculopathy with cerebral leukodystrophy are retinal vasculopathy, cognitive impairment, migraine, psychiatric abnormalities, and seizures, as well as hepatic and renal dysfunction [11, 12]. The disease is due to the *TREX1* gene and associated both with CSVD neuroimaging features and, in some cases, pseudotumoral lesions surrounded by vasogenic edema [13–15].

Finally, the cerebral autosomal recessive arteriopathy with subcortical infarcts and leukoencephalopathy (CARASIL), due to mutations in the *HTA1* gene, has been described in Asians and recently in some European cases. Clinically, the disease is characterized by recurrent lacunar strokes associated with a rapidly progressive cognitive impairment, seizures, and psychiatrics disturbance [16–20].

Although the above described single gene disorders are believed to account for a small proportion of cases, their prevalence is probably underestimated; systematic studies on well-defined phenotypes and larger series are needed since these diseases are expected to play a crucial role in our understanding of the pathogenesis of VaD and CSVD [21].

### Sporadic vascular dementia

Compared to these monogenic forms, it is reasonable to assume that sporadic VaD might have a more complex mode of inheritance, in which multiple genetic variants with small effects predispose to disease (similar to other causes of dementia). Additionally, many of the risk factors for VaD, such as hypertension, dyslipidemia, and smoking habits, are partly genetically determined and this may further complicate the picture [22]. Most of our current genetic understanding of VaD is based on candidate gene studies, while genome-wide associations studies (GWAS) have so far only contributed modestly.

#### Candidate gene studies

The candidate genes for association studies may encompass both genes implicated in stroke and Alzheimer disease and genes that affect critical biological processes to VaD. Given the multitude of candidate gene studies and the common failure of replicating the findings, we focus only on candidates that show consistent effect in two or more published studies.

From a pathophysiological view, the genes involved in lipid metabolism, especially the apolipoprotein E (*APOE*), have been the focus of genetic research over the last two decades. Several meta-analyses, including a recent one which consists of 44 studies (2481 cases, 7490 controls), found a significant association between ε4 allele carriers and increased risk of VaD, irrespective of ethnicity [23–25]. However, there was no difference in risk for VaD between ε2 and ε3 allele carriers nor in the *APOE* promoter T-427C [2]. Two polymorphisms (Q192R and L55M) of *PON1* were associated with a higher risk of VaD in Indian and French populations [26–28], but these findings for Q192R were not confirmed in two other studies on European populations [29, 30]. Several genes related to inflammation have also been related to VaD, including two polymorphisms in the *TNF-α* in Europeans [31] and Asians [32]. In addition, an association between VaD and *TGF-β1* was described in two Asian reports [33, 34]. Finally, the variant C677T of methylenetetrahydrofolate-reductase (MTHFR), which influences the level of homocysteine, was associated with VaD in Asians in three meta-analyses [35–37], and more recently also showing stronger evidence in Europeans [25].

However, candidate gene studies have not identified robust associations for various features of sporadic CSVD such as lacunar infarction, intracerebral hemorrhage, or white matter hyperintensities. Methodological issues, particularly related to inaccurate or heterogeneous phenotyping and insufficient sample sizes, have been invoked as possible reasons for this [6].

#### GWAS

To identify genetic risk factors for VaD, two GWAS have been reported, firstly, a retrospective study in Koreans (84 VaD patients, 200 controls) that did not detect any genetic association [38], and a prospective study in the Netherlands (67 patients, 5700 controls) that identified an association with the variant rs12007229 near the androgen receptor gene on the X chromosome, which was replicated in a German population (221 cases, 213 controls) [39]. However, large-scale hypothesis-free studies are lacking, thereby hampering gene discovery in VaD.

A more recent line of research has been to consider genes that have been identified through GWAS of stroke and AD. With respect to stroke, the Cohorts of the Heart and Aging Research in Genetic Epidemiology (CHARGE) consortium [40, 41], International Stroke Genetics Consortium (ISGC) [42, 43], NINDS Stroke Genetics Network (SiGN) [43], and Wellcome Trust Case Control Consortium 2 (WTCCC2) [42] have conducted separate studies totaling tens of thousands of stroke patients and hundreds of thousands of controls. A variant near *FOXF2* was found to increase risk of all-stroke, and was also associated with white matter hyperintensities. Furthermore, multiple robust associations have been reported in an apparent subtype-specific manner – *TSPAN2* was associated with large artery atherosclerosis-related stroke, *PITX2* and *ZFHX3* with cardioembolic stroke, *HDAC9* with large artery atherosclerosis stroke, and *ALDH2* with small artery stroke. However, these genes have yet to be investigated for their relation with VaD. Currently, even larger GWAS on stroke are underway. Linking all these novel genes with VaD would crucially advance our knowledge of its genetic architecture. Similarly, the International Genomics of Alzheimer’s Project has identified various genes for AD through GWAS [44], but these have yet to be associated with VaD. Interestingly though, not all these novel genes fit into known AD pathways involving amyloid or tau processing [45]. Indeed, several genes have been implicated in cardiometabolic pathways, most notably *APOE*, *CLU*, and *ABCA7*, which are related to lipid metabolism [45].

In contrast to the candidate gene studies, GWAS have recently been carried out successfully to identify novel genes underlying CSVD. For white matter hyperintensities, for instance, five loci have been identified by studying over 21,000 individuals from the CHARGE consortium [46]. Interestingly, genes at these loci are implicated in both AD and in (hemorrhagic) stroke [46]. Earlier findings have been replicated in independent population-based [47, 48] and clinical [49] samples. The CHARGE consortium also investigated whether genetic influences could be detected for the progression of white matter hyperintensities over time [50]. While a very small genetic contribution was identified, larger sample sizes and improved methods for assessing change could yield more results [50]. GWAS of MRI-defined brain infarcts have been less robust, with an identified association in the *MACROD2* gene showing inconsistent effects [51]. Nevertheless, whether the genetic associations with CSVD can be effectively reflective of those with VaD remains uncertain.

Instead of restricting genetic overlap to the top genes, an exciting alternative approach is to test for genetic overlap between clinical endpoints on a genome-wide scale. Novel methods, including linkage disequilibrium score regression [52], now make such investigations possible. Currently, a study is ongoing for stroke and AD and, if the results are positive, this will provide important evidence of a causal link between these two clinical distinct entities, which overlap clinically in the realm of VaD.

### Future developments

Despite the initial successes of GWAS, it is important to keep in mind that the explained variance of these genetic associations is limited, indicating that the largest proportion of the heritability of CSVD still remains to be elucidated. A common message from all genetic studies in the field of VaD is that we need larger sample sizes. The genetic complexity is such that there are likely thousands of unknown variants, each with a modest contribution to disease. Sufficient power can only be achieved by jointly analyzing all data and will likely even necessitate cross-consortia efforts. Furthermore, many consortia have progressed to novel technologies, such as next generation sequencing, which permit further identification of multiple causal variants, including rare variants [53].

Another important aspect of gaining power is by improving phenotype definitions, which are currently quite heterogeneous for VaD [54]. Further, mixed pathologies are common above the age of 75 and require careful statistical handling. While GWAS of clinical phenotypes has started to provide robust association with current sample sizes over hundreds of thousands of participants in VaD and other complex diseases, the endophenotype approach has shown promising results with much smaller sample sizes. The focus has so far been on white matter hyperintensities and brain infarcts, but other neuroimaging traits have yet to be analyzed. Particularly, brain microbleeds (from T2*-weighted or susceptibility-weighted imaging), enlarged perivascular spaces, and white matter integrity (from diffusion imaging) would be important phenotypes for VaD. Brain microbleeds and enlarged perivascular spaces are markers for both ischemic and hemorrhagic pathology [55, 56], and elucidating genetic factors that are common to both pathways can also shed light on VaD. Also, white matter integrity as measured by diffusion imaging provides a much more complete assessment of the burden of CSVD, since it precedes many of the ‘end stage’ findings such as white matter hyperintensities [57].

### Clinical implications of understanding the genetics of VaD

An important implication of unravelling the genetic causes of VaD would be a biological understanding of its origin. However, what genetic studies have actually demonstrated so far is that clinical definitions are rather heterogeneous; one of the primary challenges will be to create robust definitions of VaD phenotypes, along with the selection of appropriate controls and replication in independent samples [58].

Understanding the causal genes and their underlying disease mechanisms can also lead to the identification of pathways. These can provide attractive targets for intervention and subsequently stimulate drug development, for which the related field of AD provides a good example. The identification of causative mutations in *APP*, *PSEN1* and *PSEN2* in familial AD cases has led to the amyloid cascade hypothesis that changes in the *APP* gene and its processing results in the aggregation and deposition of amyloid-β, and this presumably leads to disease. In GWAS of sporadic AD, the identification of *APOE* (initially also through linkage) and, more recently, a variety of other genes that are known to be implicated in pathways such as immune response, endyctosis, and lipid metabolism may lead to novel directions in drug discovery [45].

Also, *APOE* is already used for the selection and stratification of trial participants in drug development studies for AD by using ε4 allele carriers as a high risk group. The variability in the length of an intronic poly-T repeat of the *TOMM40*, which is in linkage disequilibrium with *APOE* on chromosome 19, has been shown to also have a predictive value for disease risk [59], and an algorithm combining both *APOE* and *TOMM40* is now used in the TOMMORROW randomized control trial [60]. The identification of more genetic variants can thus lead to improved selection of persons for clinical trials.

Furthermore, the variability of response to drugs among individuals remains a key challenge of the pharmaceutical drug research and in clinical practice, both in terms of efficacy and adverse effects. This differential response of individuals is thought to be related to genetic and non- genetic factors. Pharmacogenetics (PGX) refers to the inter-individual genome-wide DNA variations and potential interactions that correlate with drug response and toxicity [61]. With the addition of new omics translational tools and technologies, the term now also encompasses alterations in gene expression and post-translational modifications (e.g., proteomics and metabolomics) that also correlate with drug response and drug toxicity. PGX studies are typically differentiated into two broad types, namely safety PGX studies and efficacy PGX studies. Safety PGX involves genes affecting drug absorption, distribution, metabolism and excretion. Variations in these genes may affect pharmacokinetic and pharmacodynamic responses that may ultimately result into adverse effects. Extensive sequencing data of such genes are increasingly being used in various discovery stages by pharmaceutical companies, as complementary tools of clinical pharmacology databases for key decision-making processes. Efficacy PGX includes studies involving genes that are known to encode “druggable” targets. Variants within these genes may affect target engagement, and thus alter efficacy. On the other hand, disease susceptibility genes, identified though large-scale GWAS analyses and meta-analyses on cases and controls, serve for the generation of new discovery hypotheses and for target validation, as well as for the selection and/or stratification of patients in phase II clinical trials. Ideally, assuming appropriate samples and ethics approvals are in place for PGX and other biomarker studies, positive efficacy signals may correlate with genetic variants in responders to the active drug and not to the placebo; these markers could then inform the much larger (and considerably more expensive) phase III trials to increase their probability of success through PGX/biomarker-enriched trial designs for the drug or several drugs at a time. Such approaches have been successfully applied in cancer, but regrettably not in most other therapy areas, including vascular dementia.

## Conclusions

Several genetic causes of monogenic forms of vascular dementia have been documented, but their prevalence is probably underestimated. Larger case series are needed to further our understanding of the pathogenesis of VaD and CSVD. For the sporadic form, there have been several replicated candidate gene studies, but unbiased genome-wide screens have remained underpowered and so far yielded only few results. Future studies will need to be larger, but could also improve power by harmonizing phenotype definitions and studying endophenotypes. Thus far, the clinical implications for VaD have been limited, especially when compared to other diseases, but will hopefully follow after more robust genetic findings in the field.
